# The Nordic maintenance care program: what are the indications for maintenance care in patients with low back pain? A survey of the members of the Danish Chiropractors' Association

**DOI:** 10.1186/1746-1340-18-25

**Published:** 2010-09-01

**Authors:** Signe F Hansen, Anne L S Laursen, Tue S Jensen, Charlotte Leboeuf-Yde, Lise Hestbæk

**Affiliations:** 1 Institute of Clinical Biomechanics, University of Southern Denmark, Odense, Denmark; 2 The Back Research Center, Ringe, University of Southern Denmark, Odense, Denmark; 3 Nordic Institute for Chiropractic and Clinical Biomechanics, University of Southern Denmark, Odense, Denmark

## Abstract

**Background:**

Maintenance care (MC) is relatively commonly used among chiropractors. However, factual information is needed on its indications for use.

**Objectives:**

This study had two objectives: 1) to describe which role patients' past history and treatment outcome play in chiropractors' decision to use MC in patients with low back pain, 2) to investigate if the chiropractors' clinical/educational background has an effect on the frequency of using MC and their indications for use of MC.

**Method:**

An anonymous questionnaire was sent to all 413 chiropractors practising in Denmark. Its main part consisted of 3 sets of 4 questions relating to one basic case of low back pain. For each case, the chiropractors were asked if they would use MC as they self-defined the term (no/perhaps/yes). There were questions also on gender, age, educational and clinical background, and on the number of MC patients seen by these chiropractors. Their decision to recommend MC was reported. Associations between the demographic variables and 1) the frequency of MC-use and 2) their indications for use of MC were tested through multivariate analysis.

**Results:**

The response rate was 72%. Non-indications for MC were: 1) a good outcome combined with no previous events, or 2) a past history of LBP and gradual worsening with treatment. Indications for MC were a good outcome combined with a previous history of low back pain between once a month and once a year. The mean proportion of MC patients per week were 22% (SD 19), ranging from 0% to 100%. The use of MC was highest among experienced chiropractors, those who were educated in North America, and clinic owners. However, in Denmark most chiropractors graduated before 1999, are educated abroad, whereas most chiropractors thereafter are educated in Denmark. Therefore, we cannot conclude whether this difference relates to education or years of experience. There were no associations detected between demographic variables and the indications for MC.

**Conclusions:**

There is relatively high consensus on when MC should and should not be used. A history of prior low back pain combined with a positive response to treatment encourages the use of MC, whereas no previous history of back pain or a worsening of symptoms discourages the use of MC. There seems to be a difference in the proportional use of MC between chiropractors with more experience educated in North America and those with less experience educated in Denmark.

## Background

Presently, it is not known if or how low back pain (LBP) can be prevented from developing. Because LBP frequently is a long-lasting or recurring problem, prevention of recurrences is as relevant as primary prevention. However, also this aspect is clouded in mystery.

Many patients with LBP will seek care from chiropractors [1,2]. Some patients will continue treatment after the acute problem has been resolved or considerably improved, because by then it appears logical to attempt to attend to the underlying cause of the recurring LBP complaint. Among chiropractors, this approach is called maintenance care (MC).

The majority of patients with LBP who are treated by chiropractors will receive spinal manipulation. Spinal manipulative therapy has been tested in a number of trials and found to be effective for LBP, at least in the short term [3,4]. Whether such treatment can prevent LBP from recurring or getting worse, however, appears only to have been tested explicitly in one pilot study, with inconclusive results [5]. Therefore, it is not known, if MC is an effective method for preventing, delaying, or mitigating recurrent episodes of LBP.

Nevertheless, this concept is fairly well accepted among chiropractors. Chiropractors in various parts of the world state that they use MC in about one third of their patients [6-9]. However, the indications for MC, as it is used today, have not been determined.

Presently, a research program is being conducted in the Nordic countries trying to illuminate the use of, the indications for, and the efficiency of MC. So far, it has been determined, that there is a large degree of consensus among Nordic chiropractors that the primary goal of MC is prevention of new episodes of LBP, although for some patients it can be prevention of deterioration [10,11]. There is also general agreement, that the two most important issues for recommending MC are frequency of episodes in the past and effect of the treatment. Thus, more previous episodes indicate a higher risk of recurrence and therefore a larger need for treatment, and there has to be a positive response to treatment if a MC-strategy is to be recommended [7,10-12]. However, despite the large degree of consensus, there is not total agreement and there are subgroups of chiropractors with different opinions.

Since prior history of LBP episodes, and positive response to treatment have been identified as commonly accepted indications for offering MC to patients, we designed this current study to explore this finding in more specific detail. We asked chiropractors in this pragmatic cross-sectional survey about their use of MC, as they self-defined the term, by presenting them with a series of clinical case scenarios that varied as to prior history (frequency and duration of the previous episodes) and response to treatment.

In addition we were curious to see, if there were other factors that would influence chiropractors' use of MC. In a previous study from Australia, it had been shown that MC was more common among practitioners who had only few new patients as compared to those who had more new patients [13]. Other factors that we thought could influence chiropractors' incentive to use MC were: gender (women perhaps being more caring than men), educational background (some chiropractic institutions being reputed for encouraging the use of MC), clinical experience (own experience might either encourage or discourage its use), and whether the respondent was a clinic owner or not (financial incentives).

This study had two objectives: 1. to describe which role patients' past history and treatment outcome play in chiropractors' attitudes to the use of MC in patients with LBP, and 2. to investigate if the chiropractors' demographic, clinical and educational background has an influence on how many patients receive MC and what type of patients are offered MC.

## Method

### The survey

A list of actively practising chiropractors was obtained from the Danish Chiropractors' Association. These were mailed a questionnaire plus a pre-stamped envelope in February 2007. In order to encourage participation, information on the study was given to participants at local chiropractic meetings across the country by two chiropractors and in the Danish chiropractors' professional journal. The questionnaires were returned anonymously.

### The questionnaire

A questionnaire was designed specifically for the purpose of this study (Additional file 1). The first page consisted of demographic questions and information on the number of MC patients as they self-defined the term. The main part of the questionnaire consisted of three separate pages each with 4 questions relating to one basic case, in which an uncomplicated patient with LBP was described. The basic case was:"A 40-year old man consults you for **low back pain **of 2 days duration with **no **additional spinal or musculoskeletal problems, and with **no **other health problems. His x-rays are **normal **for his age. There are **no **red flags and he seems to be in good shape both physically and psychologically. There are **no **aggravating factors at work or at home." The questions about this patient were constructed such that he was assumed to have received chiropractic treatment but that his past history could vary as could the outcome of the treatment.

The second page of the questionnaire related to this basic case, on this page presented as a patient who had no previous history of back problems at all but here were four different outcomes. On the third page, there was a history of recurring problems but the outcomes were the same as on the second page. On the fourth page, there were four different past histories but the outcomes were the same. Each of these 12 cases was followed by the question:"Would you consider recommending maintenance care? No/Perhaps/Yes**"**.

The questionnaire was tested a number of times among researchers and clinicians at the Back Research Center, in Ringe, Denmark, and improved to ensure face validity and user friendliness.

### Scandinavian mainstream chiropractic practice

Based on previous studies [7,10-12] and in-depths interviews with 10 Danish chiropractors (not yet published) we derived clinical parameters as operational definitions for the term"Scandinavian mainstream chiropractic practice" that we applied during our analyses and presentation of data collected in this current study. Using these operational definitions, we tried to identify those survey responses, which were most likely to be expected as answers to the 12 questions relating to the indications for the use of MC. For example, within this definition, it was considered unsuitable to offer MC to patients with no previous history of back problems at all, whereas patients with frequent past problems were considered suitable, providing that they reacted well to treatment. In 4 of the questions though (The exact wording can be seen in Additional file 1: Appendix 1: page 2 scenario 3, page 3 scenario 1 and 3, and page 4 scenario 2), the information provided was not sufficiently succinct for a clear cut decision, and therefore"mainstream" answers were based on the remaining 8 questions.

### Quality of data

For validation purposes we obtained information on all the members of the Danish Chiropractors' Association on sex, age, college of graduation, and graduation year. We compared this to the profile of our participants to establish if our study sample was representative of its target population.

The repeatability of the questionnaire was tested for two of the clinical questions by asking them twice but in different parts of the questionnaire: 1) the first scenario on the second page and fourth page, and 2) the first scenario on the third page and the third scenario on the fourth page.

The self-reported use of MC was estimated by asking for its proportional use both on the day of the study and for the last working week. Both questions were asked because the data for the present day would be easier collected (just counted) than data for the past week. However, the week-data were there in case some respondents on the day of the survey had an unusual number of MC-patients.

### Analysis and presentation of data

In the analyses, the variables age and clinical experience were collapsed into fewer categories, based on the distribution of data.

The distributions of MC use per day and per week were analyzed with the help of frequency tables and graphs and the mean value with standard deviations of the weekly proportion of MC-patients were reported. The number of"expected" answers for each participant was calculated based on the 8 predetermined"expected" answers. On the basis of the distribution of data this variable was dichotomized into two groups, consisting of"mainstream" (giving the"expected" answer for at least 7 of these 8 scenarios) vs."non-mainstream" answers (all the others).

Associations between the demographic variables and the proportional use of MC and the proportion of"mainstream" answers were tested with bivariate analyses, and thereafter with multivariate linear regression analyses for the variables found to be significant in the bivariate analyses. Because of the possibility of strong correlations between several of the demographic variables, we tested these correlations using Pearson's r. The relationship between the proportional use and the proportion of"mainstream" answers was tested using kappa statistics. Data were analysed with STATA 8.2 (STATA Corporation, 2000, *Stata Statistical Software *Release 8.2*, College Station, Tex., USA) *and a p-level of 0.05 or less was considered statistically significant.

## Results

### Description of the participants and their use of maintenance care

In all, 296 out of 413 active members of the Danish Chiropractors' Association (72%) returned their questionnaires, with an even distribution of men and women. Two-thirds of the participants were aged 30-49 years and almost half had graduated in the USA or Canada. The details are presented in Table 1 and a similar description of the members of the Danish Chiropractors' Association is presented in the same table for comparison.

**Table 1:** A comparison of the study sample in a survey of Danish chiropractors and the target population (%).

Compared variables	Danish Chiropractors' Association (N = 455, of which 413 are active members)	Study sample (N = 296)
*Gender*		
females	51	46
Males	49	45
missing		9

*Age*		
20-29	6	4
30-39	31	35
40-49	38	38
50-59	18	20
60 or more	7	4
missing		< 1

*Graduated in*		
Denmark	33	30
UK	22	24
USA/Canada	45	46
other	< 1	0
missing		< 1

*Clinical experience (yrs)*		
0-1	7	1
2-5	18	22
6-10	10	9
11-19	25	29
20 or more	38	39
missing	2	< 1

*Where do you work (several answers possible)*		
general practice	(data not available)	93
Falck health Care/Private hospital		16
Public hospital		4
Other		4

*Are you (several answers possible)*		
clinic owner	62	65
employee	38	31
both	(data not available)	3
missing		1

The mean and median values of the self-reported proportion of MC patients were almost identical for the last full working week and the day of the survey. The reported proportion of MC-patients the past week ranged from 0% to 100%, with 15 missing replies and the mean value of MC patients per week was 22% (SD19). Based on the distribution, three subgroups were determined. The first group consisted of chiropractors who defined 0% -25% of the patients seen that week as MC patients (n = 195). The second group reported that 26% -42% of their patients were MC patients (n = 46), and the third group reported their proportion of MC patients to be 43%-100% (n = 40). For ease of reporting, these groups were called"low","medium", and"high", respectively.

### Validity of data

The study sample closely resembled the target population, i.e. all the members of the Danish Chiropractors' Association as seen in Table 1.

The internal consistency was good for one pair of identical questions (an acute patient with no previous history of LBP and good response to treatment: page 1, scenario 1 and page 3, scenario1) with agreement in 94% of the cases. However, the second pair of identical questions (a patient with recurrent LBP, one episode per month for five years and good response to treatment: page 2, scenario 1 and page 3, scenario 3) was less convincing with 54% total agreement.

### Indications for use of maintenance care

In relation to the indications for the use of MC, 40% (n = 118) of the participants were classified as"mainstream" practitioners, giving the expected answer in at least 7 of the 8 cases. For each of the 8 individual cases, 57% to 89% of the participants responded as expected.

#### 1. Maintenance care in patients with no previous history of back problems but with different outcomes

Overall, the results of the first set of cases were in accordance with the expectations of the research group, as the majority of respondents found it unsuitable to provide MC to patients with no previous history of LBP at all. There was only one possible exception. In scenario 3, depicting a patient who does not improve over two months of treatment ("some days are good, some are bad"), 54% of the chiropractors responded either"perhaps" or"yes" to the consideration of MC. For this scenario, the research team expected the majority to reply"no" (Table 2).

**Table 2:** The use of maintenance care in patients with no previous history of low back pain and different outcomes of treatment.

	"Would you consider recommending MC to this patient?"			
	**No**	**Perhaps**	**Yes**	**Missing**

**Follow-up scenarios**	**Frequency** **(%)**	**Frequency** **(%)**	**Frequency** **(%)**	**Frequency** **(%)**

You treat him once and the symptoms disappear directly after you manipulated the painful area. You follow for him two months and the pain does not reappear, the movement pattern is normal, and you cannot provoke any symptoms by palpation or other tests.	***264*** ***(89%)***	16 (5%)	12 (4%)	4 (1%)

You treat him once and the symptoms disappear directly after you manipulated the painful area. You follow him for two months and after one month there is a recurrence. But after one more treatment the pain does not reappear, the movement pattern is normal, and you cannot provoke any symptoms by palpation or other tests.	***169*** ***(57%)***	82 (28%)	39 (13%)	6 (2%)

You treat him for two months and you can see from his file that some days are good some are bad, but in all there is no difference really.	**132** **(45%)**	92 (31%)	67 (23%)	5 (2%)

You treat him for 2 months and he is getting gradually worse.	***260*** ***(88%)***	18 (6%)	13 (4%)	5 (2%)

The following basic case was given:"A 40-year old man consults you for low back pain of 2 days duration with no additional spinal or musculoskeletal problems, and with no other health problems. His x-rays are normal for his age. There are no red flags and he seems to in good shape both psychically and psychologically. There are no aggravating factors at work or at home." The expected answers are written in bold, and where the majority of the practitioners agree with the research team, the answer is also in italics.

The use of maintenance care based on patients with a history of one episode of LBP per month the last 5 years lasting 5-6 days resolving spontaneously and different outcomes.

#### 2. Maintenance care in patients with monthly episodes of LBP but with different outcomes

Information collected in this part of the questionnaire did not always agree with the expectations of the research group (Table 3). In the first two scenarios, in which the patient is getting better in relation to the treatment, the"expected" answer would be to consider MC. However, in the patient who improves directly on the first visit and whose pain does not recur within two months, only 41% would offer MC, whereas 58% would offer MC to the other person who had a recurrence after one month. If the patient does not improve after two months ("some days are good some are bad"), 54% of the chiropractors answer"yes" or"perhaps" to offering MC. In this case the research team had expected a response of"no". For the patient who gradually gets worse, the research team had also anticipated a majority response of"no" and 88% of the respondents agreed with that concept.

**Table 3:** The use of maintenance care based on patients with a history of one episode of LBP per month the last 5 years lasting 5-6 days resolving spontaneously and different outcomes.

	"Would you consider recommending MC to this patient?"			
	**No**	**Perhaps**	**Yes**	**Missing**

**Follow-up scenarios**	**Frequency** **(%)**	**Frequency** **(%)**	**Frequency** **(%)**	**Frequency** **(%)**

You treat him once and the symptoms disappear directly after you manipulated the painful area. You follow him for two months and the pain does not reappear, the movement pattern is normal, and you cannot provoke any symptoms by palpation or other tests.	83 (28%)	88 (30%)	**120** **(41%)**	5 (2%)

You treat him once and the symptoms disappear directly after you manipulated the painful area. You follow him for two months and after one month there is a recurrence. But after one more treatment the pain does not reappear, the movement pattern is normal, and you cannot provoke any symptoms by palpation or other tests.	27 (9%)	94 (32%)	***171*** ***(58%)***	4 (1%)

You treat him for two months and you can see from his file that some days are good some are bad, but in all there is no difference really.	**129** **(44%)**	90 (30%)	71 (24%)	6 (2%)

You treat him for 2 months and he is gradually getting worse.	***259*** ***(88%)***	21 (7%)	10 (3%)	6 (2%)

The following basic case was given:"A 40-year old man consults you for low back pain of 2 days duration with no additional spinal or musculoskeletal problems, and with no other health problems. His x-rays are normal for his age. There are no red flags and he seems to in good shape both psychically and psychologically. There are no aggravating factors at work or at home." The expected answers are written in bold, and where the majority of the practitioners agree with the research team, the answer is also in italics.

#### 3. Maintenance care in patients with good outcome but different past histories

As expected, 87% would not offer MC to a patient with immediate and lasting positive response and no previous history of back pain. The opinions were mixed if there had been 1-2 episodes of LBP a year, with 46% answering"perhaps", whereas the research team's expected answer was"yes". The majority, however, agreed that MC would be suitable for more frequent past episodes. For monthly episodes, 70% were in favour, and 69% for weekly episodes. This was in line with the"expected" answers (Table 4).

**Table 4:** The use of maintenance care in patients with different past histories but identical outcomes.

	"Would you consider recommending MC to this patient?"			
	**No**	**Perhaps**	**Yes**	**Missing**

**Past history**	**Frequency** **(%)**	**Frequency** **(%)**	**Frequency** **(%)**	**Frequency** **(%)**

He has **never **previously had any back pain at all.	***257*** ***(87%)***	19 (6%)	12 (4%)	8 (3%)

Over the past 5 years he has had **1-2 episodes of LBP a year**, each event lasting **5-6 days **and resolving spontaneously.	67 (23%)	135 (46%)	**86** **(29%)**	8 (3%)

Over the past 5 years he has had **1 episode of LBP per month**, each event lasting **5-6 days **and resolving spontaneously.	20 (7%)	61 (21%)	***207*** ***(70%)***	8 (3%)

Over the past 5 years he had about **1 episode per week**, each event lasting **2-3 days **and resolving spontaneously.	29 (10%)	54 (18%)	***205*** ***(69%)***	8 (3%)

The following course of treatment was given: You treat him once and the symptoms disappear directly after a manipulation to the painful area. You follow him for two months and the pain does not reappear, the movement pattern is normal and you cannot provoke any symptoms by palpation or other tests. The expected answers are written in bold, and where the majority of the practitioners agree with the research team, the answer is also in italics.

### Associations between the demographic background and the proportional use of maintenance care

There were no statistically differences in the use of MC between men and women or between age groups. However, there were statistically significant differences between the proportion of MC-patients per week and the other factors investigated: 1) graduates from USA/Canada had the highest use of MC and graduates from Denmark the lowest, 2) more years in practice was associated with a higher use of MC, and 3) clinic owners used MC more often than employees, see Tables 5, 6, 7. However, these three factors are closely interrelated. Almost all chiropractors in Denmark with less than 11 years of experience are graduates from Denmark. Because of the strong correlation between graduation and experience (Pearson's r = 0.82, p < 0.001), graduation and employment (Pearson's r = -0.54, p < 0.001), and between experience and employment (Pearson's r = -0.57, p < 0.001) we did not perform a multi-variate analysis. It is therefore not possible to determine whether high use of MC is determined by educational background or experience.

**Table 5:** The weekly use of MC among Danish chiropractors in relation to clinical experience.

	Use of MC per week			
**Clinical experience (years)**	**Low**	**Medium**	**High**	**Total**

**0-1**	3 (100)	0	0	3 (100)

**2-5**	54 (86)	5 (8)	4 (6)	63 (100)

**6-10**	19 (73)	4 (15)	3 (12)	26 (100)

**11-19**	49 (58)	23 (27)	12 (14)	84 (100)

**20 or more**	69 (66)	14 (13)	21 (20)	104 (100)

**Total**	194 (69)	46 (16)	40 (14)	280 (100)

Test for linear trend, p < 0.005

**Table 6:** The weekly use of MC among Danish chiropractors in relation to country of graduation.

	Use of MC per week			
**Graduated in**	**Low**	**Medium**	**High**	**Total**

Denmark	73 (84)	8 (9)	6 (7)	87 (100)

UK	46 (71)	14 (22)	5 (8)	65 (100)

USA/Canada	76 (59)	24 (19)	29 (22)	129 (100)

Total	195 (69)	46 (16)	40 (14)	281 (100)

Pearson chi2(4) = 20.2974 Pr = 0.000

**Table 7:** The weekly use of MC among Danish chiropractors in relation to employment.

	Use of MC per week			
**Employement**	**Low**	**Medium**	**High**	**Total**

Clinic owner	116 (63)	34 (19)	33 (18)	183 (100)

Employee	72 (81)	10 (11)	7 (8)	89 (100)

Both	6 (75)	2 (25)	0	8 (100)

Total	194 (69)	46 (16)	40 (14)	280 (100)

Pearson chi2(4) = 10.5322 Pr = 0.032

### Associations between the demographic background, care and"mainstream" use of MC

Neither the demographic data nor the use of MC was associated with the"mainstream" answers in the analyses.

## Discussion

The results of this study indicate that our study sample was representative of the membership of the Danish Chiropractors' Association. Among Danish chiropractors there is a wide variation in the use of MC; some not using it at all and others always. A strong determinant for this was, not surprisingly, their educational background, with the American-educated chiropractors being more likely to have more MC patients than those educated in the UK or Denmark. It is possible that the use of MC is a major differential factor between European-style and American-style chiropractic. One should therefore be careful when interpreting data on this topic from different parts of the world. However, since there is a strong correlation between country of graduation and years of clinical experience among Danish chiropractors, this difference could also be related to experience.

Whether MC is a useful approach or not, is not known, but assuming that it does have an effect, it should be used in such a way as to be cost-effective for the patient. Therefore it seemed logical that good outcome should be a major prerequisite, as should a considerable risk of future LBP. This was also the result in this study as well as in previous studies.

Interestingly, respondents who provided the unexpected answers could do this in both directions, i.e. not offering MC to a case with a profile assumed to be suitable or offering MC to those assumed to be unsuitable. This shows there is some diversity in the chiropractic profession regarding MC. If such a thing as"correct" answers exists to these survey questions, some chiropractors will offer MC more frequently and others less frequently. On that note, it must be remembered that the clinical scenarios presented in the survey have very limited information and many other factors (e.g. the psychological profile of the patient, various types of spinal problems, etiology of the back pain etc.) might influence the chiropractor's choice [11]. Despite these shortcomings of the survey, there is a large degree of consensus among the chiropractors.

There seems to be a general agreement that MC should be used in patients at risk for future problems and that the past history is a predictor of that. Further, the patient should improve with treatment. As for the cut-off point for past LBP events, it lies somewhere between one episode per year and one per month, but probably closer to the latter.

For clinicians, also the non-indications for MC treatment are important. Almost 90% agreed that patients who recovered very quickly, remained stable over two months, and who had no past history of LBP should not have MC. And about as many of our participants would not consider MC for patients who got gradually worse.

Curiously, there was no majority"no"- response for the patients who"oscillated" (some days good some days bad) with no real improvement. Also according to previous Swedish and Danish studies, subgroups of respondents who would recommend MC also in patients who do not report a clinically relevant amount of improvement were noted [7,10,11]. Further, in a Finnish study, some argued that lack of treatment success could also be an indication for MC [12]. We had hoped for some clear indications on this issue, but some confusion remains. Perhaps such answers arise because some chiropractors consider chiropractic treatment always to be of benefit, regardless the reporting of symptoms. This was neither the opinion of the research team, nor of the majority of respondents, but until the value of MC has been tested in a number of randomized controlled clinical trials, nobody can tell who is right.

## Conclusions

We were able to reach four distinct conclusions:

• About 2/3 of the Danish chiropractors reported to use MC on between 0 and 25% of their patients.

• The most frequent use of MC was reported by chiropractors who were graduates from North America, experienced chiropractors and clinic owners.

• For Danish chiropractors in general, the indications for MC in patients with LBP were: good short-term outcome and at least 1-2 previous episodes per year.

• There were no associations between the demographic background and the indications for the use of MC.

## Conflict of interests

The authors declare that they have no competing interests.

## Authors' contributions

SHF and ALSH designed the study, collected and analyzed the preliminary data and wrote a report on part of the results as a part requirement for their masters degree in Health Sciences (Biomechanics), supervised by CLY and LH. TSJ supervised and assisted with the data analysis. CLY and LH were responsible for the final manuscript. All authors read and approved the final manuscript.

## Supplementary Material

Additional file 1 **Questionnaire**. A copy of the questionnaire used in the survey about the use of maintenance care in Danish chiropractic practice. Click here for file
