# Supplementary material for: The Nordic maintenance care program: what are the indications for maintenance care in patients with low back pain? A survey of the members of the Danish Chiropractors' Association
Source: Chiropr Osteopat. 2010 Sep 1;18:25. doi: 10.1186/1746-1340-18-25 (PMC2940898; doi:10.1186/1746-1340-18-25)
Supplement: Additional file 1 — Questionnaire. A copy of the questionnaire used in the survey about the use of maintenance care in Danish chiropractic practice. [file 1746-1340-18-25-S1.DOC]

**App. 1: TREATMENT STRATEGIES-QUESTIONNAIRE, DENMARK 2007**

Please draw a ring around the correct answer

**SEX:** *Male Female*

**AGE:** *20-29 30-39 40-49 50-59 60 or more*

**GRADUATED IN:**

*Denmark UK USA/Canada Other*

**CLINICAL EXPERIENCE (Years):**

*0-1 2-5 6-10 20 or more*

**WHERE DO YOU WORK (Several answers possible):**

*Private practice Falck Health Care/Private hospital Public hospital Other*

**ARE YOU (Several answers possible):**

*Clinic owner Employee Both*

**YOUR USE OF MAINTENANCE CARE (MC):**

*Number of MC patients last full working day / Total number of visits last full working day x 100 = _________%*

*Number of MC patients last full working week / Total number of visits last full working week x 100 = _________%*

**Below, in the box, you will find a basic case. Underneath are 4 different follow-up scenarios. We would like to know for which, if any, of these 4 follow-up scenarios, you would consider recommending maintenance care.**

The basic case is:

*A 40-year old man consults you for* ***low back pain*** *of 2 days duration with* ***no*** ***additional spinal or musculoskeletal problems****, and with* ***no other health problems****.*

*His X-rays are* ***normal*** *for his age.*

*There are* ***no red flags*** *and he seems to be in good shape both physically and psychologically. There are* ***no aggravating factors*** *at work or at home.*

In addition, in relation to the questions below: He has **no previous history of back problems at all.**

A. The 4 different follow-up scenarios are:

(For each of the scenarios below, please draw a ring around the best answer)

1. You treat him once and the symptoms disappear directly after you manipulated the painful area. You follow him for two months and the pain does not reappear, the movement pattern is normal, and you cannot provoke any symptoms by palpation or other tests. *Would you recommend maintenance care?*

*No Perhaps Yes*

2. You treat him once and the symptoms disappear directly after you manipulated the painful area. You follow him for two months, and after one month there is a recurrence. But after one more treatment the pain does not reappear, the movement pattern is normal and you cannot provoke any symptoms by palpation or other tests. *Would you recommend maintenance care?*

*No Perhaps Yes*

3. You treat him for two months and you can see from his file that some days are good and others are bad, but in all there is no difference really. *Would you recommend maintenance care?*

*No Perhaps Yes*

4. You treat him for two months and he is getting gradually worse. *Would you recommend maintenance care?*

*No Perhaps Yes*

**Below are the same scenarios and the basic case is still the same**:

*A 40-year old man consults you for* ***low back pain*** *of 2 days duration with* ***no*** ***additional spinal or musculoskeletal problems****, and with* ***no other health problems****.*

*His X-rays are* ***normal*** *for his age.*

*There are* ***no red flags*** *and he seems to be in good shape both physically and psychologically. There are* ***no aggravating factors*** *at work or at home.*

**The difference is** that in relation to the questions below, over the past 5 years he has had **1 episode per month**, each event lasting **5-6 days** and resolving spontaneously.

A. The 4 different follow-up scenarios are:

(For each of the scenarios below, please draw a ring around the best answer)

1. You treat him once and the symptoms disappear directly after you manipulated the painful area. You follow him for two months and the pain does not reappear, the movement pattern is normal, and you cannot provoke any symptoms by palpation or other tests. *Would you recommend maintenance care?*

*No Perhaps Yes*

2. You treat him once and the symptoms disappear directly after you manipulated the painful area. You follow him for two months, and after one month there is a recurrence. But after one more treatment the pain does not reappear, the movement pattern is normal and you cannot provoke any symptoms by palpation or other tests. *Would you recommend maintenance care?*

*No Perhaps Yes*

3. You treat him for two months and you can see from his file that some days are good and others are bad, but in all there is no difference really. *Would you recommend maintenance care?*

*No Perhaps Yes*

4. You treat him for two months and he is getting gradually worse. *Would you recommend maintenance care?*

*No Perhaps Yes*

**Below you will find 4 different past histories, still for the same basic case, as described in the box on the previous pages. This time all have the same course of treatment (see the box below) BUT 4 different past histories. We would like to know for which, if any, of these past histories, you would consider recommending maintenance care.**

The course of treatment is:

You treat him **once** and the symptoms **disappear directly** after a manipulation to the painful area. You follow him for **two months** and the pain **does not reappear**, the movement pattern is **normal** and you **cannot provoke** any symptoms by palpation or other tests.

B. The 4 different past histories are:

1. He has **never** previously had any back pain at all. *Would you recommend maintenance care to this patient?*

*No Perhaps Yes*

2. Over the past 5 years he has had **1-2 episodes of LBP a year**, each event lasting **5-6 days** and resolving spontaneously. *Would you recommend maintenance care to this patient?*

*No Perhaps Yes*

3. Over the past 5 years he has had **1 episode per month**, each event lasting **5-6 days** and resolving spontaneously. *Would you recommend maintenance care to this patient?*

*No Perhaps Yes*

4. Over the past 5 years he has had about **1 episode per week**, each event lasting **2-3 days** and resolving spontaneously. *Would you recommend maintenance care to this patient?*

*No Perhaps Yes*
